# In vitro antifungal resistance profile of *Candida* strains isolated from Saudi women suffering from vulvovaginitis

**DOI:** 10.1186/s40001-019-0399-0

**Published:** 2020-01-04

**Authors:** Mohamed T. Yassin, Ashraf A. Mostafa, Abdulaziz A. Al-Askar, Rashad Bdeer

**Affiliations:** 1 Botany and Microbiology Dept., College of Science, King Saud University, P.O. 2455, Riyadh, 11451 Saudi Arabia; 2 Microbiology Department, Regional Laboratory at King Saud Medical City, P.O. 2897, Riyadh, 11196 Saudi Arabia

**Keywords:** *Candida* vaginitis, Phylogenetic analysis, Antifungal agents, Resistance

## Abstract

**Background:**

Vulvovaginal candidiasis (VVC) represents a universal health hazard that contributes to significant morbidity in women. Resistance of *Candida* to antifungal therapy has been reported as a public health problem. So, the objective of our current study is to detect resistance profile of different candidal strains.

**Methods:**

In this study, isolated *Candida* strains were identified by conventional methods, confirmed by internal transcribed spacer (ITS) sequencing, and phylogenetically analyzed with reference strains in GenBank. Also, sensitivity of different Candida strains to common antifungal agents was evaluated by disc diffusion method.

**Results:**

*Candida albicans* was identified as the most frequent strain (63%) followed by non-*albicans* strains, such as *C. glabrata* (20%), *C. tropicalis* (13%), and *C. krusei* (4%). Sensitivity of *Candida* strains (*C. albicans*, *C. tropicalis* and *C. glabrata*) to commonly used antifungal agents was evaluated through the disc diffusion method. *C. glabrata* was the most resistant strain and considered to be a multidrug-resistant pathogen, while both, *C. albicans* and *C. tropicalis* showed high susceptibility to terbinafine. In contrast, *C. albicans* showed resistance to fluconazole, clotrimazole, and nystatin, while *C. tropicalis,* considered as the most sensitive strain, was susceptible to all the antifungal agents tested except nystatin. Terbinafine was the most effective antifungal agent against both *C. tropicalis* and *C. albicans,* and hence its minimum inhibitory concentration (MIC) and minimum fungicidal concentration (MFC) for *C. albicans* and *C. tropicalis* were evaluated. MICs of terbinafine against *C. albicans* and *C. tropicalis* were 5 μg/ml and 2.5 μg/ml, while their MFCs were 10 μg/ml and 5 μg/ml, respectively.

**Conclusion:**

The emergence of resistant *Candida* strains necessitates conduction of the antifungal susceptibility test prior to deciding the medication regime.

## Background

*Candida albicans,* a commensal microorganism, is a part of the normal flora on mucosal surfaces of the human body such as the gastrointestinal, respiratory and genitourinary tracts [1]. *Candida* species constitute 20–50% of the normal flora colonizing the female genital tract with *C. albicans*, the causative agent of vaginal candidiasis, being predominant (about 80.5%) followed by *Candida glabrata* [2–4]. Establishment of fungal infection by the *Candida* sp. is mediated through virulence factors like transition between yeast and hyphal forms, formation of biofilms, secretion of hydrolytic enzymes and expression of invasion and adhesion proteins [5]. Hydrolytic enzymes such as hemolytic enzymes, lipases and phospholipases produced by the *Candida *sp. contribute to its virulence while the secreted aspartyl proteinases play a role in adherence, penetration and invasion of host tissues, inducing tissue damage, thereby aiding the establishment of infection [6, 7]. Vulvovaginal candidiasis is characterized by multiple symptoms such as dyspareunia, pruritis, itching, soreness, and vaginal erythema, and affects majority of the women during their lifetime. The use of antibiotics, diabetes mellitus, pregnancy and immunodeficiency are all risk factors that disturb the vaginal microflora enabling the establishment of vaginal infection [8, 9]. In addition, establishment of vulvovaginal candidiasis infection occurs due to many behavioral and host-dependent factors such as the use of oral contraceptives, sexual activity and other hygiene habits [10]. Significant morbidity in women worldwide due to recurrent vulvovaginal candidiasis (RVVC) caused by *C. albicans* has been reported [11]. Many of these infections may cause high mortality rates due to the development of resistance against antifungal agents [12]. Fluconazole is currently being used as an effective therapy to control RVVC, but the development of antibiotic resistant strains necessitates the discovery of new drugs [13]. Advent of multidrug-resistant *C. albicans* strains may lead to invasive candidiasis increasing the mortality and morbidity in hospitals [14]. Recently, incidence of vulvovaginal candidiasis in pregnant woman has been recorded to be significantly more than that in non-pregnant woman (28.2% and 7.9%, respectively) [15]. Similar results were confirmed by Bauters et al. [16], who reported vulvovaginal candidiasis incidence of 32% in pregnant and 19.3% in non-pregnant women. Nystatin exhibited a high antifungal activity against all the organisms tested while resistance against azole antifungal drugs varied between species. Only one strain of *C. glabrata* showed resistance to clotrimazole and fluconazole while others exhibited dose-dependent susceptibility to fluconazole [17]. A study conducted by Salehei et al. [18] showed isolated vaginal *Candida* strains to be highly susceptible to terbinafine, clotrimazole and miconazole, but resistant to fluconazole and econazole. Antifungal sensitivity test also indicated that clotrimazole was the most effective antifungal agent against 68 of the isolated *Candida* strains followed by nystatin (51) and fluconazole (29) [19]. The objective of our current study was to isolate *Candida* strains from vaginitis patients in Riyadh region and identify the most dominant strains. In addition, susceptibility of different strains to antifungal agents commonly used in treatment of vulvovaginal candidiasis was also evaluated.

## Methods

### Isolation of microorganisms causing vaginitis infection

Examination of a total of 394 vaginal swabs from pregnant (138) and non-pregnant women (256) suffering from vaginitis, collected from the Regional Laboratory at King Saud Medical City during June 2016 to June 2018, was performed. All the swabs were subjected to wet mount examination by rolling them on glass slides with one drop of saline solution (0.85%) for detection of *Trichomonas vaginalis* (the causative agent of trichomoniasis). The swabs were also subjected to Gram staining for the detection of bacterial vaginosis infections. The swabs were cultured on Sabouraud dextrose agar (SDA) medium supplemented with chloramphenicol (0.5 g/l), incubated at 37 °C for 48–72 h to isolate *Candida* vaginitis strains. All vaginal swabs were also cultured on mannitol salt agar, MacConkey agar, and blood agar for the isolation of Gram-negative and Gram-positive infectious bacterial strains.

### Identification of isolated *Candida* strains

The isolated strains were preliminarily identified according to their cultural, microscopic, and chemical characteristics. The *Candida albicans* and non-*albicans* strains were differentiated by culturing them in CHROM agar medium as well as performing the germ tube test by inoculating *Candida* strains in human serum (0.5 ml) followed by incubation at 37 °C for 3 h. The identification of isolated *Candida* strains was confirmed using the API20C AUX kit and internal transcribed spacer (ITS) sequencing technique.

### Molecular identification of concerned *Candida* strains

The identification of the isolated *C. albicans* strain and two non-*albicans* strains was confirmed by bidirectional sequencing technique. Genomic DNA was extracted using GeneJET Genomic DNA Purification Kit K0721 (Thermo Fisher Scientific, US). Polymerase chain reaction (PCR) using universal forward and reverse primers of ITS1 (5ʹ-TCC GTA GGT GAA CCT GCG G-3ʹ) and ITS4 (5ʹ-TCC TCC GCT TAT TGA TAT GC-3ʹ), respectively, was performed to amplify the ITS1-5.8S-ITS2 domain. Bidirectional sequencing of PCR products was performed by Macrogen (Korea). The obtained sequences were blasted to compare with reference strains in GenBank. The sequences were submitted to GenBank (https://www.ncbi.nlm.nih.gov/genbank/) and their corresponding accession numbers were obtained. Comparison of sequences of isolated vaginal strains with the reference strains was achieved using MEGA 7 software and phylogenetic tree was built using the neighbor-joining method.

### Antifungal susceptibility test

The disc diffusion method was used to check the sensitivity of the isolated vaginal candida strains (*Candida albicans, C. tropicalis, C. glabrata*) to different antifungal agents. Reference strains, *Candida albicans* (ATCC 18804), *Candida glabrata* (ATCC 15545), and *Candida tropicalis* (ATCC 13803) were used for quality control purposes. Antifungal drugs, namely, fluconazole (Pfizer, UK), terbinafine (Novartis, Switzerland), itraconazole (Janssen, Belgium), clotrimazole, and nystatin (Sigma Aldrich, USA) were dissolved in methanol using an ultrasound sonicator. The three *Candida* strains were subcultured onto SDA slants and incubated at 37 °C for 48 h. Microbial suspensions were prepared by harvesting the *Candida* growth in 5 ml of sterile saline water and the absorbance was adjusted to 30% at 560 nm using a spectrophotometer. The yeast cells were enumerated at the corresponding absorbance by the plate count technique, and the viable cell count was 10^7^ CFU/ml for each *Candida* strain. Fifteen milliliter of SDA medium was sterilized, poured into sterile plates (as a basal layer), followed by 10 ml of seeded medium previously inoculated with microbial suspension (1 ml of 10^7^ CFU/100 ml of medium) to obtain 10^5^ CFU for each ml of the medium. Sterile filter paper discs (8 mm) were loaded with different antifungal drugs mentioned before, at the following concentrations (25, 50, 50, 20 and 50) µg/disc, respectively. Antifungal discs were placed over seeded layer plates and incubated at 37 °C for 48 h. The inhibition zone diameters measured using Vernier calipers were considered to indicate the sensitivity of different vaginal *Candida* strains to different antifungal agents. The results were interpreted according to Clinical and Laboratory Standards Institute (CLSI) guidelines for the detection of resistant, dose-dependent, and sensitive *Candida* strains [20].

### Determination of minimum inhibitory concentration (MIC) of the most effective antifungal drug (terbinafine) against isolated *Candida* vaginitis strains

Minimum inhibitory concentration is defined as the lowest concentration of antifungal agents against common *Candida* strains that inhibits their growth after 48 h of incubation. The most effective antifungal drug (terbinafine), which showed strong antimicrobial activity, was investigated to determine its MIC and to evaluate its efficiency in controlling *Candida* strains causing vaginitis. A disc diffusion method was used in which 10 ml of SDA medium was poured into sterile petri dishes as a base layer followed by 15 ml of medium seeded with microbial inoculum previously prepared (1 ml of 10^7^ CFU of *Candida* suspension/100 ml of culture medium) to obtain a final concentration of 10^5^ CFU/ml of medium. Petri dishes were allowed to solidify and sterile filter paper discs (8 mm) loaded with different concentrations of terbinafine (1.25, 2.5, 5, 10, 20, 40 µg/ml) were placed on it. The plates were refrigerated for 2 h to allow terbinafine diffusion throughout the medium, followed by incubation at 37 °C for 48 h. Inhibition zone diameters were measured using Vernier calipers and recorded against the concentration of terbinafine.

### Determination of minimum fungicidal concentrations (MFCs) of the most effective antifungal drug (terbinafine)

Minimum fungicidal concentration (MFC) is defined as the lowest concentration of antifungal agent showing microbicidal activity, i.e., no microbial growth. MFC is an indicator of the dosage required for complete eradication of *Candida* growth. Streaks were taken from inhibition zones of MIC concentration and two other successive concentrations, and subcultured onto freshly prepared SDA plates. The plates were incubated at 35 °C for 48 h and examined for microbial growth.

## Results

### Sample collection and preliminary identification

About 205 vaginal swabs from the 394 clinical samples tested positive for vaginitis infection. Incidence of vaginitis in pregnant and non-pregnant women was 65.9% and 44.5%, respectively, as seen in Table 1. *Candida* vaginitis was the main cause of vaginal infections (58.5%), followed by bacterial vaginosis (41%) and trichomoniasis infections (0.5%). The prevalence of vulvovaginal candidiasis was higher in both, pregnant (64.8%) and non-pregnant women (53.5%), while bacterial vaginosis was more frequently observed in non-pregnant women than in pregnant women, as seen in Table 2. The isolated *Candida* vaginitis strains were preliminarily identified in order to determine the most predominant disease-causing strains. About 166 isolates of *Candida* vaginitis strains and 87 bacterial vaginosis strains were isolated, as shown in Table 3. High incidence of vaginal infections among pregnant (57.1%) and non-pregnant (34.2%) women in the age group of 26–35 years was observed (refer Table 4).

**Table 1:** Positive vaginitis infections among pregnant and non-pregnant women

Vaginal infections	Pregnant women		Non-pregnant women		Total patients no.	
	No.	Incidence %	No.	Incidence %	No.	Incidence %
Positive	91.0	65.9	114.0	44.5	205.0	52.0
Negative	47.0	34.1	142.0	55.5	189.0	48.0
Total	138.0	–	256.0	–	394.0	100.0

**Table 2:** Incidence of *Candida* vaginitis, bacterial vaginosis and trichomoniasis in pregnant and non-pregnant women

Patients	*Candida* vaginitis		Bacterial vaginosis		Trichomoniasis		Total patients no.	
	No.	Incidence%	No.	Incidence%	No.	Incidence%	No.	Incidence%
Pregnant	59	64.8	32	35.2	0	0.0	91	44.4
Non-pregnant	61	53.5	52	45.6	1	0.9	114	55.6
Total	120	58.5	84	41.0	1	0.5	205	100.0

**Table 3:** Characterization of vaginal microflora isolated from vaginitis patients

Vaginitis infection	*Candida* vaginitis		Bacterial vaginosis				Trichomoniasis	
			Gram −ve		Gram +ve			
	No.	%	No.	%	No.	%	No.	%
Single	59.0	35.50	26.0	29.90	12.0	27.90	1.0	100.0
Mixed	107.0	64.50	61.0	70.10	31.0	72.10	0.0	0.00
Total	166.0	–	87.0	–	43.0	–	1.0	–

**Table 4:** Relation between age groups and positive culture swabs in pregnant and non-pregnant women

Age groups (years)	Pregnant women		Non-pregnant women		Total patients no	
	No.	Incidence%	No.	Incidence%	No.	Incidence%
25 <	15	16.5	32	28.1	47	22.9
26–35	52	57.1	39	34.2	91	44.4
36–45	24	26.4	22	19.3	46	22.4
46–55	0	0.0	15	13.1	15	7.3
56 >	0	0.0	6	5.3	6	2.9
Total no.	91	44.4	114	65.9	205	100.0

### Identification of isolated *Candida* strains

Primary identification of the isolated *Candida* strains was achieved by germ tube test, CHROM agar and API20C AUX which revealed *Candida albicans* to be the most predominant strain (63%) followed by *C. glabrata* (20%), *C. tropicalis* (13%) and *C. krusei* (4%).

### Molecular identification of concerned *Candida* strains

**Fig. 1:**
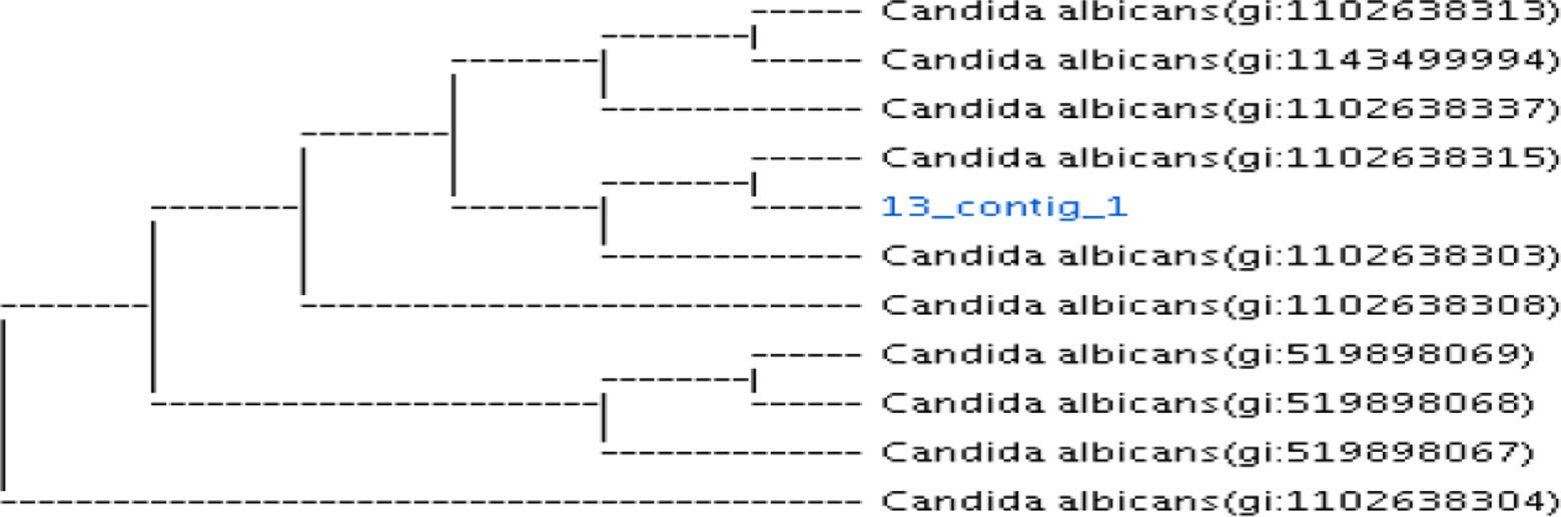
Phylogenetic analysis of isolated vaginal *Candida albicans* strain with other reference strains in GenBank compiled using neighbor-joining method. *13-contig-1 is the isolated *C. albicans* vaginal strain with Accession number MK300693 submitted to GenBank

**Fig. 2:**
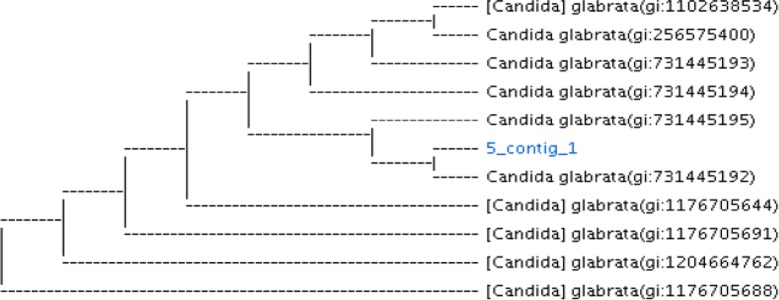
Phylogenetic analysis of isolated vaginal non-albicans strain (*C. glabrata*) with other reference strains in GenBank compiled using neighbor-joining method. *5-contig-1 is the isolated *C. glabrata* vaginal strain with Accession number MK300697 submitted to GenBank

**Fig. 3:**
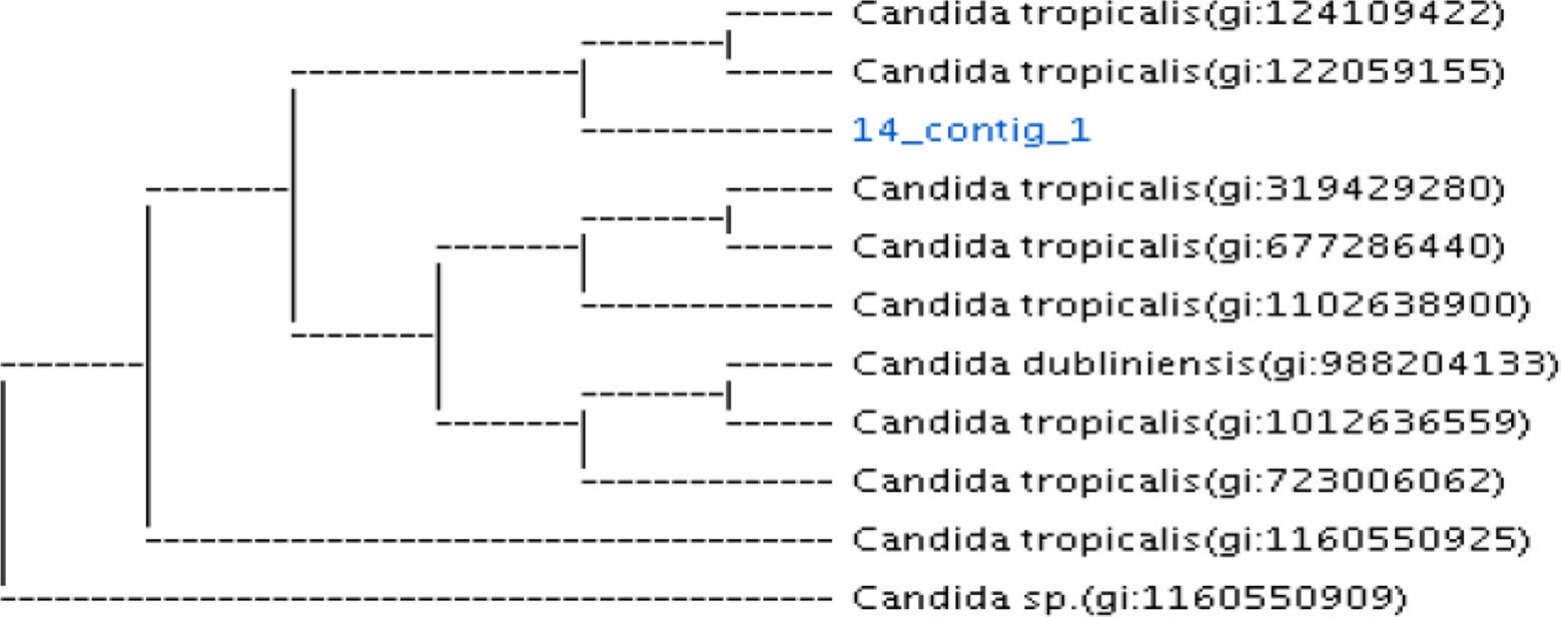
Phylogenetic analysis of isolated vaginal non-albicans strain (*C. tropicalis*) with other reference strains in GenBank compiled using neighbor-joining method. *14-contig-1 is the *C. tropicalis* isolated vaginal strain with Accession number MK300695 submitted to Genbank

Molecular identification and the phylogenetic analysis of the isolated vaginal *Candida* strains using ITS sequencing technique as a confirmatory test for fungal identification was performed. The *Candida albicans* strain, non-*albicans* strain, *C. tropicalis* and *C. glabrata* with Accession numbers (MK300693), (MK300695), and (MK300697), respectively, were submitted to GenBank. *Candida albicans* strain showed 100% similarity with *Candida* reference strain no. (KY101885) while *C. tropicalis* showed 100% similarity with candida reference strains no. (KX977559, KX944465, KY102470, and KU950724) in GenBank. *C. glabrata* revealed 100% similarity to reference strains of Accession numbers (KP131705 and HG970737). Phylogenetic trees of the three isolated candida strains (*C. albicans*, *C. glabrata* and *C. tropicalis*) with reference strains in GenBank can be seen in Figs. 1, 2, 3, respectively.

### Antifungal susceptibility test

Disc diffusion method was used to evaluate resistance of *C. albicans* and non-*albicans* strains (*C. tropicalis*, *C. glabrata*) to common antifungal drugs. Antifungal sensitivity test revealed that *C. albicans* was highly sensitive to terbinafine and itraconazole drugs exhibiting inhibition zone diameters of 27.2 and 17.9 mm, respectively, while its resistance to clotrimazole, nystatin and fluconazole was interpreted according to CLSI guidelines (refer Table 5). *Candida tropicalis* showed resistance to nystatin, but was highly sensitive to terbinafine, fluconazole and clotrimazole, exhibiting inhibition zone diameters of 24.4, 24.1 and 21.2 mm, respectively, as seen in Table 6. Its sensitivity to itraconazole was dose dependent. *Candida glabrata* showed resistance to all antifungal drugs used in the current study.

**Table 5:** The chemical classes and susceptibility criteria of the used antifungal agents according to CLSI

Antifungal agents	Conc. (µg/disc)	Chemical classes	Inhibition zone diameter (mm)		
			Resistant	Dose dependent	Sensitive
Clotrimazole	50	Azoles	≤ 11	12–19	≥ 20
Fluconazole	25		≤ 16	15–18	≥ 19
Itraconazole	50		≤ 9	10–15	≥ 16
Nystatin	20	Polyenes	≤ 16	17–24	≥ 25
Terbinafine	50	Allylamines and thiocarbamates	≤ 11	12–19	≥ 20

**Table 6:** Antimicrobial susceptibility test of the isolated *Candida* vaginitis strains against different antifungal drugs

Antifungals	Conc. (μg/ml)	Inhibition zone diameter (mm) of the isolated vaginal candida strains		
		*C. albicans*	*C. tropicalis*	*C. glabrata*
Clotrimazole	50	11.07 ± 0.43	21.20 ± 0.64	0.00 ± 0.00
Fluconazole	25	13.67 ± 0.09	24.10 ± 0.12	0.00 ± 0.00
Itraconazole	50	17.93 ± 0.38	15.50 ± 0.46	0.00 ± 0.00
Nystatin	20	12.73 ± 0.49	18.60 ± 0.12	15.53 ± 0.66
Terbinafine	50	27.27 ± 0.08	24.40 ± 0.15	11.73 ± 0.91

All data are a mean of triplicates ± standard error

### Determination of minimum inhibitory concentration (MIC) and minimum fungicidal concentration (MFC)

**Fig. 4:**
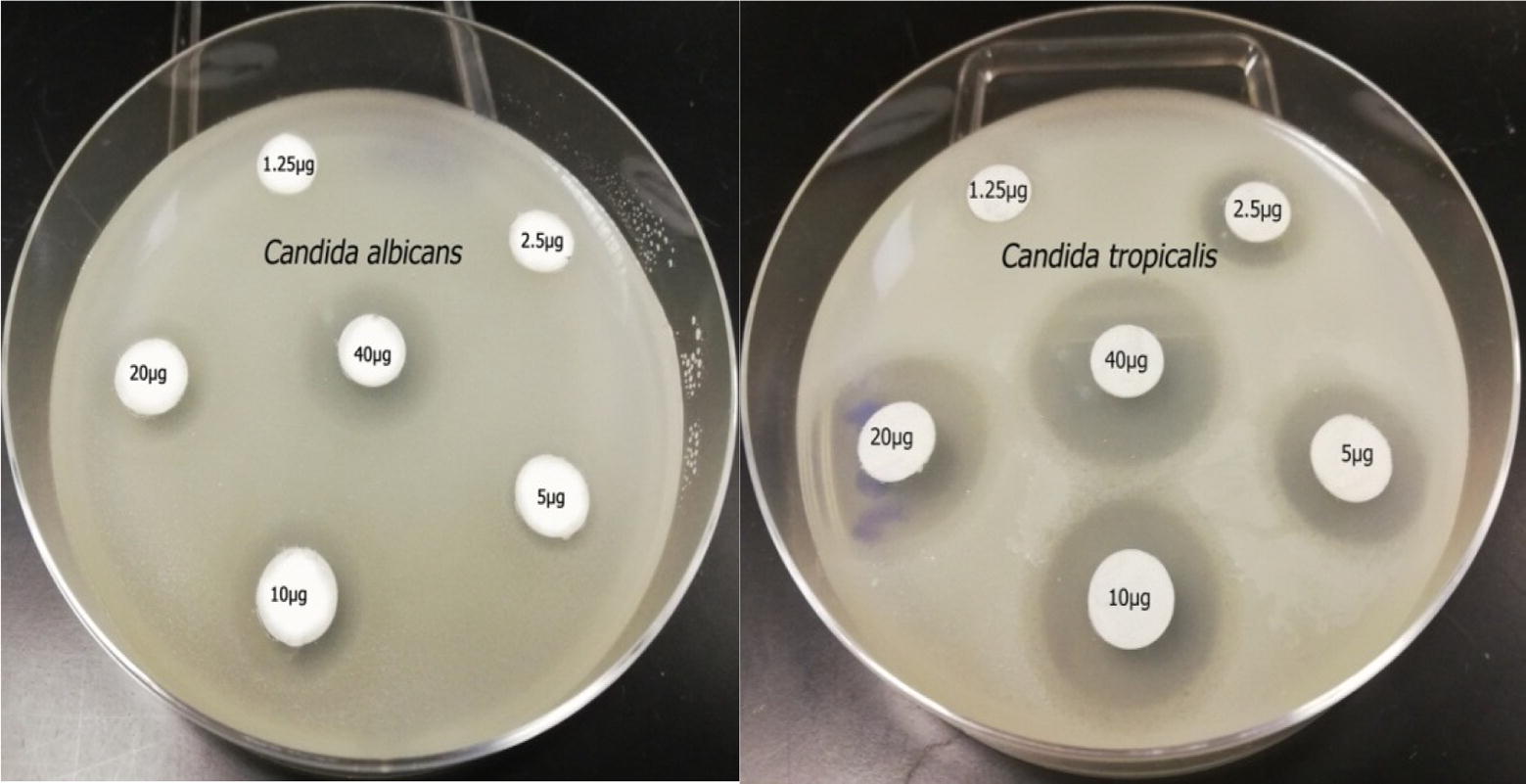
MIC of the most effective antifungal drug (terbinafine) against *C. albicans* and *C. tropicalis*

Terbinafine was identified to be the most effective antifungal drug against *C. albicans* and *C. tropicalis* with MIC values of 5 and 2.5 μg/ml and inhibition zone diameters of 9.2 and 11.2 mm, respectively (refer Table 7). *Candida tropicalis* was more sensitive to terbinafine compared to *C. albicans* as shown in Fig. 4. MFC of terbinafine against *C. albicans* was 10 μg/ml while it was 5 μg/ml for *C. tropicalis*. MFC results confirmed that *C. tropicalis* was more susceptible to terbinafine than *C. albicans*.

**Table 7:** MICs of terbinafine as the most effective antifungal drug

Terbinafine Conc. (μg/ ml)	Inhibition zone diameter (mm) of vaginal *Candida* strains	
	*C. albicans*	*C. tropicalis*
1.25	0.00 ± 0.00	0.00 ± 0.00
2.50	0.00 ± 0.00	11.10 ± 0.06
5.00	9.20 ± 0.06	11.50 ± 0.05
10.0	12.80 ± 0.17	14.60 ± 0.17
20.0	14.20 ± 0.17	15.40 ± 0.12
40.0	17.23 ± 0.13	18.87 ± 0.15

## Discussion

Approximately, 58.5% of the women examined in this study suffered from vulvovaginal candidiasis episodes. This result is in accordance with that of Kamath et al. [21] who recorded 47.7% of pregnant women to be infected with vulvovaginal candidiasis and the disease incidence in non-pregnant woman to be 20.3%. The high infection rate among pregnant woman may be attributed to higher secretion of sex hormones during pregnancy, especially during the last trimester [16, 21, 22]. The present study identified *C. albicans* to be the predominant causative agent of vulvovaginal candidiasis disease (63%) followed by non-*albicans* strains *C. glabrata* (20%) and *C. tropicalis* (13%). Our study results are in agreement with that of Amouri et al. [23], who ascertained that *C. albicans* represented the predominant strain (76.3%) followed by *C. glabrata* (19.3%) and *C. tropicalis* (1.4%) among the strains causing vulvovaginal candidiasis. Preliminary identification of vaginal yeast strains from vulvovaginal candidiasis patients showed that *C. glabrata* represented the second most dominant strain and this coincides with the results obtained by Richter et al. [12], Mahmoudabadi et al. [24] and Hedayati et al. [25]. Internal transcribed spacer (ITS) sequencing is considered as a rapid and accurate tool for identification of fungal pathogens [26]. Genetic variation within ITS region is considered to be sufficient for identification and typing of different fungal strains [27–29]. Azoles and allylamines (terbinafine) act as antifungal agents by inhibition of ergosterol biosynthesis while polyenes (nystatin) disrupt cell functions by binding to ergosterol in plasma membrane [30, 31]. Moreover, allylamines disrupt synthesis of ergosterol synthesis through inhibition of squalene epoxidase enzyme [32]. Resistance of *C. albicans* to fluconazole antifungal drug was recorded in current study as seen in Fig. 4 and this may be due to its use for long-term treatment [33, 34]. Similar result was obtained by Scocozza et al. [35] who reported the resistance of *C. albicans* strains to fluconazole. *C. glabrata* showed high resistance to azole drugs similar to that observed in previous studies recorded by Oxman et al. [36] and Pfaller et al. [37]. Resistance of *C. albicans* and *C. glabrata* to azole antifungal drugs may be due to several factors including the induction of drug efflux pumps and sequestration of antifungal agents [38–40]. *C. albicans* and *C. tropicalis* resistance to nystatin has also been previously demonstrated in studies conducted by Khan et al. [19]. *C. tropicalis* isolated from infected patients show high sensitivity to terbinafine and clotrimazole and these results were in accordance with the results of Salehi et al. [18]. Terbinafine was highly effective against *C. albicans and C. tropicalis* strains with MIC values 2.5 and 5 µg/ml, respectively, as shown in Table 7. Similar results have been reported by several researchers and hence terbinafine is the first drug of choice in treatment of infections caused by dermatophytes [41, 42]. Antifungal resistance of *C. glabrata* to all antifungal agents tested in the current study may be attributed to its ability to form biofilms [43].

## Conclusions

Terbinafine was the most effective therapeutic agent against isolated *C. albicans* and *C. tropicalis* strains. Also, we can conclude that performing antifungal sensitivity test in hospitals prior to start of medical therapy is essential owing to the high emergence of multidrug-resistant Candida strains.

## Data Availability

The datasets used and/or analyzed during the current study are available from the corresponding author on reasonable request.

## Abbreviations

VVC: vulvovaginal candidiasis
RVVC: recurrent vulvovaginal candidiasis
ATCC: American Type Culture Collection
ITS: internal transcribed spacer
CFU: colony forming unit
MIC: minimum inhibitory concentration
MFC: minimum fungicidal concentration

## References

[1] Kashem SW, Igyártó BZ, Gerami-Nejad M, Kumamoto Y, Mohammed J, Jarrett E, Drummond RA, Zurawski SM, Zurawski G, Berman J (2015). *Candida albicans* morphology and dendritic cell subsets determine T helper cell differentiation. Immunity.

[2] Grigoriou O, Baka S, Makrakis E, Hassiakos D, Kapparos G, Kouskouni E (2006). Prevalence of clinical vaginal candidiasis in a university hospital and possible risk factors. Eur J Obstet Gynecol Reprod Biol.

[3] Pierce CG, Lopez-Ribot JL (2013). Candidiasis drug discovery and development: new approaches targeting virulence for discovering and identifying new drugs. Expert Opin Drug Discov.

[4] Wang F-J, Zhang D, Liu Z-H, Wu W-X, Bai H-H, Dong H-Y (2016). Species distribution and in vitro antifungal susceptibility of vulvovaginal Candida isolates in China. Chin Med J.

[5] Berman J, Sudbery PE (2002). *Candida albicans*: a molecular revolution built on lessons from budding yeast. Nat Rev Genet.

[6] Silva S, Negri M, Henriques M, Oliveira R, Williams DW, Azeredo J (2012). *Candida glabrata, Candida parapsilosis* and *Candida tropicalis*: biology, epidemiology, pathogenicity and antifungal resistance. FEMS Microbiol R.

[7] Schaller M, Borelli C, Korting HC, Hube B (2005). Hydrolytic enzymes as virulence factors of *Candida albicans*. Mycoses.

[8] Sobel JD (2007). Vulvovaginal candidosis. Lancet.

[9] Johal HS, Garg T, Rath G, Goyal AK (2016). Advanced topical drug delivery system for the management of vaginal candidiasis. Drug Deliv.

[10] Gonçalves B, Ferreira C, Alves CT, Henriques M, Azeredo J, Silva S (2016). Vulvovaginal candidiasis: epidemiology, microbiology and risk factors. Crit Rev Microbiol.

[11] Talaei Z, Sheikhbahaei S, Ostadi V, Hakemi MG, Meidani M, Naghshineh E, Yaran M, Naeini AE, Sherkat R (2017). Recurrent vulvovaginal candidiasis: could it be related to cell-mediated immunity defect in response to Candida antigen?. Int J Fertil Steril.

[12] Richter SS, Galask RP, Messer SA, Hollis RJ, Diekema DJ, Pfaller MA (2005). Antifungal susceptibilities of *Candida* species causing vulvovaginitis and epidemiology of recurrent cases. J Clin Microbiol.

[13] Sobel JD (2016). Recurrent vulvovaginal candidiasis. Am J Obstet Gynecol.

[14] Dadar M, Tiwari R, Karthik K, Chakraborty S, Shahali Y, Dhama K (2018). *Candida albicans*-biology, molecular characterization, pathogenicity, and advances in diagnosis and control—an update. Microb Pathog.

[15] Neerja J, Aruna A, Paramjeet G (2006). Significance of Candida culture in women with vulvovaginal symptoms. J Obstet Gynecol India.

[16] Bauters TG, Dhont MA, Temmerman MI, Nelis HJ (2002). Prevalence of vulvovaginal candidiasis and susceptibility to fluconazole in women. Am J Obstet Gynecol.

[17] Nejat ZA, Farahyar S, Falahati M, Khozani MA, Hosseini AF, Faiazy A (2018). Molecular identification and antifungal susceptibility pattern of non-albicans *Candida* species isolated from vulvovaginal candidiasis. Iran Biomed J.

[18] Salehei Z, Seifi Z, Mahmoudabadi A (2012). Sensitivity of vaginal isolates of *Candida* to eight antifungal drugs isolated from Ahvaz, Iran.. Jundishapur J Microbiol.

[19] Khan M, Ahmed J, Gul A, Ikram A, Lalani FK (2018). Antifungal susceptibility testing of vulvovaginal *Candida* species among women attending antenatal clinic in tertiary care hospitals of Peshawar. Inf Drug Resist.

[20] Clinical and Laboratory Standards Institute. Reference method for antifungal disk diffusion susceptibility testing of yeasts; approved guideline. CLSI document M44-A. CLSI, Wayne, PA: Clinical Laboratory Standards Institute 19087–1898, USA. 2004.

[21] Kamath P, Pais M, Nayak MG (2013). Risk of vaginal candidiasis among pregnant women. Int J Curr Microbiol Appl Sci.

[22] Nelson M, Wanjiru W, Margaret MW (2013). Prevalence of vaginal candidiasis and determination of the occurrence of *Candida* species in pregnant women attending the antenatal clinic of Thika District Hospital, Kenya. Open J Med Microbiol.

[23] Amouri I, Sellami H, Borji N, Abbes S, Sellami A, Cheikhrouhou F (2011). Epidemiological survey of vulvovaginal candidosis in Sfax, Tunisia.. Mycoses.

[24] Mahmoudabadi AZ, Najafyan M, Alidadi M (2010). Clinical study of *Candida* vaginitis in Ahvaz, Iran and susceptibility of agents to topical antifungal. Pak J Med Sci.

[25] Hedayati MT, Taheri Z, Galinimoghadam T, Aghili SR, Cherati JY, Mosayebi E (2015). Isolation of different species of Candida in patients with vulvovaginal candidiasis from Sari, Iran. Jundishapur J Microbiol.

[26] Iwen PC, Hinrichs S, Rupp M (2002). Utilization of the internal transcribed spacer regions as molecular targets to detect and identify human fungal pathogens. Med Mycol.

[27] Chen YC, Eisner JD, Kattar MM, Rassoulian-Barrett SL, LaFe K, Yarfitz SL (2000). Identification of medically important yeasts using PCR-based detection of DNA sequence polymorphisms in the internal transcribed spacer 2 region of the rRNA genes. J Clin Microbiol.

[28] De Baere T, Claeys G, Swinne D, Massonet C, Verschraegen G, Muylaert A (2002). Identification of cultured isolates of clinically important yeast species using fluorescent fragment length analysis of the amplified internally transcribed rRNA spacer 2 region. BMC Microbiol.

[29] Li Y, Leaw S, Chen J-H, Chang H, Chang T-C (2003). Rapid identification of yeasts commonly found in positive blood cultures by amplification of the internal transcribed spacer regions 1 and 2. Eur J Clin Microbiol Infect Dis.

[30] Antonovics J, Abbate JL, Baker CH (2007). Evolution by any other name: antibiotic resistance and avoidance of the E-word. PLoS Biol.

[31] Cowen LE (2008). The evolution of fungal drug resistance: modulating the trajectory from genotype to phenotype. Nat Rev Microbiol.

[32] Niewerth M, Korting H-C (2000). The use of systemic antimycotics in dermatotherapy. Eur J Dermatol.

[33] Shahid Z, Sobel JD (2009). Reduced fluconazole susceptibility of *Candida albicans* isolates in women with recurrent vulvovaginal candidiasis: effects of long-term fluconazole therapy. Diagn Microbiol Infect Dis.

[34] Khan F, Baqai R (2010). In vitro antifungal sensitivity of fluconazole, clotrimazole and nystatin against vaginal candidiasis in females of childbearing age. J Ayub Med Coll Abbottabad.

[35] Scocozza L, Azula N, Córdoba S, Smayevsky J, Relloso M (2018). High level of fluconazole resistance in *Candida* spp. isolated from vaginal specimens in adults women in a Universitary Hospital in Buenos Aires, Argentina. Int J Infect Dis..

[36] Oxman DA, Chow JK, Frendl G, Hadley S, Hershkovitz S, Ireland P (2010). Candidaemia associated with decreased in vitro fluconazole susceptibility: is *Candida* speciation predictive of the susceptibility pattern?. J Antimicrob Chemother.

[37] Pfaller MA, Jones RN, Castanheira M (2014). Regional data analysis of *Candida* non-*albicans* strains collected in United States medical sites over a 6-year period, 2006–2011. Mycoses.

[38] Mukherjee PK, Chandra J, Kuhn DM, Ghannoum MA (2003). Mechanism of fluconazole resistance in *Candida albicans* biofilms: phase-specific role of efflux pumps and membrane sterols. Inf Immun.

[39] Ramage G, Mowat E, Jones B, Williams C, Lopez-Ribot J (2009). Our current understanding of fungal biofilms. Crit Rev Microbiol.

[40] Fanning S, Mitchell AP (2012). Fungal biofilms. PLoS Pathog.

[41] Shivakumar V, Okade R, Rajkumar V, Sajitha K, Prasad S (2011). Intermittent pulse-dosed terbinafine in the treatment of *tinea corporis* and/or *tinea cruris*. Indian J Dermatol.

[42] Barot BS, Parejiya PB, Patel HK, Gohel MC, Shelat PK (2012). Microemulsion-based gel of terbinafine for the treatment of onychomycosis: optimization of formulation using D-optimal design. AAPS PharmSciTech.

[43] Rodrigues CF, Silva S, Henriques M (2014). *Candida glabrata*: a review of its features and resistance. Eur J Clin Microbiol Infect Dis.

